# Electroacupuncture Attenuates CFA-Induced Inflammatory Pain by Regulating CaMKII

**DOI:** 10.1155/2020/8861994

**Published:** 2020-12-31

**Authors:** Yixiao Gu, Shuangdong Chen, Yunchang Mo, Yingying Tu, Na Chen, Xiaoyong Zhao, Shan Li, Qimin Yu, Qinxue Dai, Junlu Wang

**Affiliations:** ^1^Department of Anesthesiology, The First Affiliated Hospital of Wenzhou Medical University, Wenzhou, 325035 Zhejiang, China; ^2^Department of Anesthesiology, Taizhou Hospital of Zhejiang Province Affiliated to Wenzhou Medical University, Taizhou, Zhejiang, China; ^3^Wencheng County People's Hospital, Wenzhou, Zhejiang, China

## Abstract

Ca^2+^/calmodulin-dependent protein kinase II (CaMKII) is a multifunctional serine/threonine kinase that is ubiquitously distributed in the central and peripheral nervous systems. Moreover, its phosphorylated protein (P-CaMKII) is involved in memory, mood, and pain regulation in the anterior cingulate cortex (ACC). Electroacupuncture (EA) is a traditional Chinese therapeutic technique that can effectively treat chronic inflammatory pain. However, the CaMKII-GluA1 role in EA analgesia in the ACC remains unclear. This study investigated the role of P-CaMKII and P-GluA1 in a mouse model of inflammatory pain induced by complete Freund's adjuvant (CFA). There were increased P-CaMKII and P-GluA1 levels in the ACC. We found that intracerebroventricular injection of KN93, a CaMKII inhibitor, as well as EA stimulation, attenuated complete Freund's adjuvant-induced pain behavior. Further, EA increased pCaMKII-PICK1 complex (abbreviated as C-P complex) levels. Our findings demonstrate that EA inhibits inflammatory pain by inhibiting CaMKII-GluA1 phosphorylation. P-CaMKII is involved in EA analgesia as the pCaMKII-PICK1 complex.

## 1. Introduction

Inflammatory pain is a common condition that is difficult to clinically treat. It involves both cellular and noncellular immunity [1, 2]. Moreover, its clinical characteristics include decreased pain threshold, enhanced pain response, and spontaneous pain [3]. There is a need to identify effective therapeutic methods that have minimal side effects. According to the theory of Chinese medicine, there are hundreds of acupuncture points in the human body; acupuncture points, especially the Zusanli and Sanyinjiao points, can be stimulated to relieve pain [4, 5]. The World Health Organization has reported that acupuncture can treat 77 diseases [6]. Moreover, electroacupuncture (EA) is widely used in clinical practice [7–10].

The anterior cingulate cortex (ACC) is involved in high cortical functions, including nociception, chronic pain, cognition, and emotion [11]. The ACC is activated by various noxious stimuli. Moreover, it is involved in cognitive processes and plays a crucial role in pain modulation [12–14], specifically the endogenous analgesic system [15]. EA on Zusanli and Sanyin acupoints in a formalin-induced inflammatory pain rat model has been shown to have an analgesic effect; this effect, obtained by contralateral electroacupuncture, disappears after ACC damage [16]. Ca^2+^/calmodulin-dependent protein kinase II (CaMKII) is a multifunctional serine/threonine kinase, which is usually composed of 12 subunits that are each encoded by one of four genes (*α*, *β*, *γ*, and *δ*) [17].CaMKII is crucially involved in synaptic plasticity and long-term potentiation (LTP) [18]. Inhibition of spinal CaMKII expression has been shown to prevent thermal hyperalgesia and mechanical allodynia [19, 20]. The alpha-amino-3-hydroxy-5-methyl-4-isoxazole propionic acid (AMPA) receptor mediates substantial excitatory transmission in the brain. AMPA receptors are heteromultimeric and are assembled from several subunits, including GluA1, GluA2, GluA3, and GluA4 [21]; specifically, in the forebrain, it mainly consists of GluA1 and GluA2 [22]. Notably, the GluA1 subunit is crucially involved in acute, chronic, or persistent inflammatory responses [23, 24]. GluA1- and GluA2-containing AMPA receptors have high and lowCa^2+^ permeability, respectively [25, 26]. Previous studies have shown that GluA1 and CaMKII play a key role in the LTP process and plasticity changes; a nociceptive stimulus induces neuronal sensitization in the spinal and supraspinal structures, similarly to LTP [27–29]. Moreover, PICK1 is a protein containing PDZ and BAR domains [30], and previous studies have shown that the catalytic domain of CaMKII is colocalized with the PICK1 BAR domain in heterologous cells and neurons [31]. However, it remains unclear whether the CaMKII-GluA1 pathway and C-P complex are involved in inflammatory pain regulation in the ACC. Moreover, the electrical regulation of this pathway has not been established. Furthermore, the mechanisms underlying EA involvement in this pathway remain unclear.

In this study, we used a mouse model of inflammatory pain induced by complete Freund's adjuvant (CFA). We hypothesized that the CaMKII-GluA1 pathway is involved in the inflammation-induced pain process in the ACC. Additionally, we hypothesized that the EA analgesia mechanism could be associated with the CaMKII pathway in the ACC.

## 2. Methods

### 2.1. Animal

In this study, we obtained male C57BL/6 mice (age range: 6-8 weeks old; weight range: 18-20 g) from the Beijing Vital River Laboratory Animal Technology Co., Ltd. (Beijing, China). The mice were housed under controlled temperature conditions (22°C) and a 12 h light/dark cycle with ad libitum access to food and water at the Experimental Animal Center of the Wenzhou Medical University. The number of animals in each group is 4 in the experimental group (*n* = 4) and 7 in each group in behavior test (*n* = 7). Mice were randomly grouped. We performed all behavior tests during the light cycle between 10 : 00 h and 14 : 00 h. The experimental protocol was approved by the Institutional Animal Care and Use Committee of Wenzhou Medical University (Wenzhou, Zhejiang, China).

### 2.2. Inflammatory Pain Model and Electroacupuncture Simulation

Complete Freund's adjuvant (CFA, 25 *μ*l, Sigma-Aldrich) was used to model inflammatory pain; as controls, the Sham group of mice received subcutaneous injections of PBS (25 *μ*l, pH = 7.4).

EA stimulation was conducted as described previously. Mice were anesthetized with 1% sevoflurane during EA stimulation sessions [32]. After mice were deeply anesthetized, the skin of acupuncture areas was shaved, and the skin was disinfected. EA stimulation: mice in the EA group received a 30-minute session of electroacupuncture (2-15 Hz alternating wave, 1.0 mA) every 2 days for 1 week (7 days) [32], with acupuncture needles (Yunlong, China), being applied at Zusanli (ST36) and Sanyinjiao (SP6) in the right hind leg. Sham electroacupuncture (SEA) was acupuncture needle insertion into ST36 and SP6 points without current stimulation or manual needle manipulation.

### 2.3. Behavioral Testing

EA stimulation started 2 hours after CFA modeling (day 1), and behavioral tests were performed 2-3 hours after EA stimulation. Behavioral testing was conducted as described previously [32]. Paw withdrawal responses to mechanical stimuli were measured with a von Frey monofilament (0.16 g; North Coast Medical, Inc., Gilroy, CA, USA). All mice were in special Plexiglas cubicles for 1 h. A set of von Frey fibers was used to assess the mechanical sensitivity (0.008, 0.02, 0.04, 0.07, 0.16, 0.4, 0.6, 1, 1.4, 2, and 4) and observe the mice withdrawal response. A positive reaction was considered as toe spread, rapid paw withdrawal, and paw licking. This experiment shows that a 0.16 g stimulus is the closest to the 50% paw withdrawal threshold value. We used percentage to evaluate pain intensity: number of paw withdrawals/10 trials. Thermal hyperalgesia was determined with a Model 336 analgesia meter (IITC, Inc., Life Science Instruments, Woodland Hills, CA, USA). Mice were acclimated in Plexiglas cubicles for 1 h. When the mouse was quiet, a heating light source was pointed at the plantar surface of the hind paw; the time taken by the mouse to retract its feet paw is the mouse withdrawing latency. The measurements were repeated five times at 5 min intervals for each paw and then averaged and analyzed.

### 2.4. Western Blot and Coimmunoprecipitation

The mice were euthanized under 3-4% sevoflurane anesthesia. Under deep anesthesia, the mice were transcardially perfused with ice-cold saline solution. Next, the ACC tissue was quickly removed and stored at –80°C for subsequent analysis. Subsequently, it was lysed in radioimmunoprecipitation buffer (Solarbio, China) containing phenylmethanesulfonyl fluoride (Solarbio, China) and phosphatase inhibitor cocktail (Sigma-Aldrich, USA) and homogenized through ultrasonic agitation. We determined the protein concentration using a Bicinchoninic Acid Protein kit (Thermo Fisher, USA). We used 500 *μ*g of total protein for coimmunoprecipitation. We added 20 *μ*l of Protein A/G Agarose and 2.5 *μ*l of normal mouse IgG in each tube and incubated it with rocking for 1 hat 4°C. Next, the agarose beads were removed via centrifugation, and 2 *μ*g of primary antibodies (anti-PICK1, 75-040, NeuroMab, Davis, CA) was added and incubated overnight with rocking at 4°C. On the next day, we removed the agarose beads via centrifugation and washed them thrice with lysis buffer. We added 1X SDS-PAGE sample loading buffer to each tube and maintained it at 100°C for 3 min in a metal bath. Regarding Western blotting, 20 *μ*g of the sample was loaded where the proteins were separated through SDS-PAGE and transferred to a polyvinylidene fluoride membrane (0.45 *μ*m, Bio-Rad, USA). Subsequently, the membranes were incubated with 5% low-fat milk in TBST for 1 h at room temperature (20-25°C). Next, the membrane was incubated overnight with the following primary antibodies: anti-GAPDH (AP0063, BioWorld; St. Louis Park, MN, USA), anti-CaMKII (Ab52476, Abcam, UK), anti-phospho-CaMKII-Thr286 (Ab124880, Abcam, UK), anti-GluA1 (Ab109450, Abcam, UK), and anti-phospho-GluA1 (Ser831) (Ab109464, Abcam, UK). The membranes were then incubated for 1 h at room temperature with a secondary antibody. Finally, the membranes were developed using an Excellent Chemiluminescent Substrate kit (Thermo Fisher, USA), and the bands were quantified using Image Lab analysis software (Bio-Rad Laboratories).

### 2.5. Immunohistochemistry Staining

The mouse ACC tissue was fixed with 4% paraformaldehyde for 24 h, and ACC tissue sections (4 *μ*m) were cut from paraffin-embedded tissue blocks, followed by incubation with the anti-phospho-CaMKII (Ab124880, Abcam, UK) overnight at 4°C. The following day, the sections were washed three times with PBS and then stained with DAB for 15 s (a two-step kit, PV9001, ZSGB-BIO, Beijing, China).

### 2.6. Immunofluorescence Staining

The sections were fixed with 4% paraformaldehyde and blocked with 5% donkey serum for 1 h at room temperature, followed by incubation with the anti-phospho-CaMKII (sab4504356, Sigma-Aldrich, USA) overnight at 4°C. The next day, the sections were washed three times with PBS and incubated with a secondary antibody DyLight 488 goat anti-rabbit IgG (1 : 200, Jackson) for 1 h at room temperature. Nuclei were stained with DAPI and imaged with Leica fluorescent microscope.

### 2.7. Intracerebroventricular Injection

We administered intracerebroventricular injections as previously described [33]. The mice were anesthetized using isoflurane and placed on a small animal stereotaxic apparatus (Shenzhen Ward Life Science and Technology Co., Ltd., Shenzhen, China). A small hole was drilled in the skull (AP,-0.5 mm; ML, -1.0 mm; DV, -2.0 mm; stereotactic coordinates corresponding to the mouse brain atlas) [34]. Next, the mice were injected with KN93 (1 *μ*l, 1 mM, Sigma-Aldrich, USA) and an equal volume of KN92 using a 10 *μ*l Hamilton syringe [35]. After suturing the scalp, the mice were placed on a homeothermic blanket to recover from anesthesia and then returned to their cages.

### 2.8. Statistical Analysis

All statistical analyses were conducted using SPSS20.0 software. All data are presented as the mean ± standard error of the mean. Western blotting and behavioral test results were analyzed by one-way and two-way repeated analysis of variance, respectively. Statistical significance was set at *P* < 0.05.

## 3. Results

### 3.1. EA Effects on CFA-Induced Hyperalgesia

**Figure 1:**
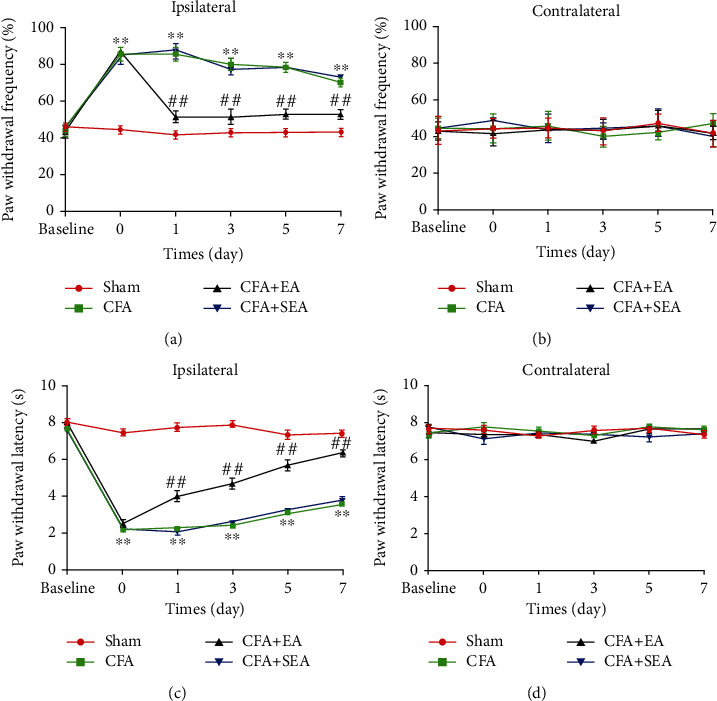
Effects of EA on CFA-induced mechanical and thermal pain threshold (*n* = 7/group). The paw withdrawal threshold was tested at base, 1 day, 3 days, 5 days, and 7 days after CFA injection. (a) The paw withdrawal frequency of the CFA group was increase, compared with the Sham group on the ipsilateral side. EA stimulation significantly decreased the paw withdrawal frequency. Sham EA has no effect on paw withdrawal frequency. (c) The paw withdrawal latency of the CFA group was increased, compared with the Sham group on the ipsilateral side. EA stimulation significantly increased the paw withdrawal latency. Sham EA has no effect on paw withdrawal latency. (b, d) No difference among the 4 groups on the contralateral side. ^∗^*P* < 0.05, ^∗∗^*P* < 0.01 vs. sham group; ^#^*P* < 0.05, ^##^*P* < 0.01 vs. CFA group.

Measurement of paw withdrawal latency (PWL) and frequency (PWF) in the ipsilateral hind paws of mice revealed CFA-induced hyperalgesia and antihyperalgesic effects of EA.  Figure 1 presents the behavioral experiment results. Before CFA injection, there was no significant between-group difference in the baseline PWF and PWL of the ipsilateral hind paws. Compared with the Sham group, the CFA group had a significantly lower mechanical and thermal pain threshold of the right hind paw on days 1, 3, 5, and 7. Compared with the CFA group, the EA group had significantly higher mechanical and thermal pain threshold values. There were no significant decreases in the contralateral side. These findings indicate that EA on Zusanli acupoints can relieve CFA-induced inflammatory pain.

### 3.2. EA Reduces P-CaMKII and P-GluA1 Protein Expression in the ACC

**Figure 2:**
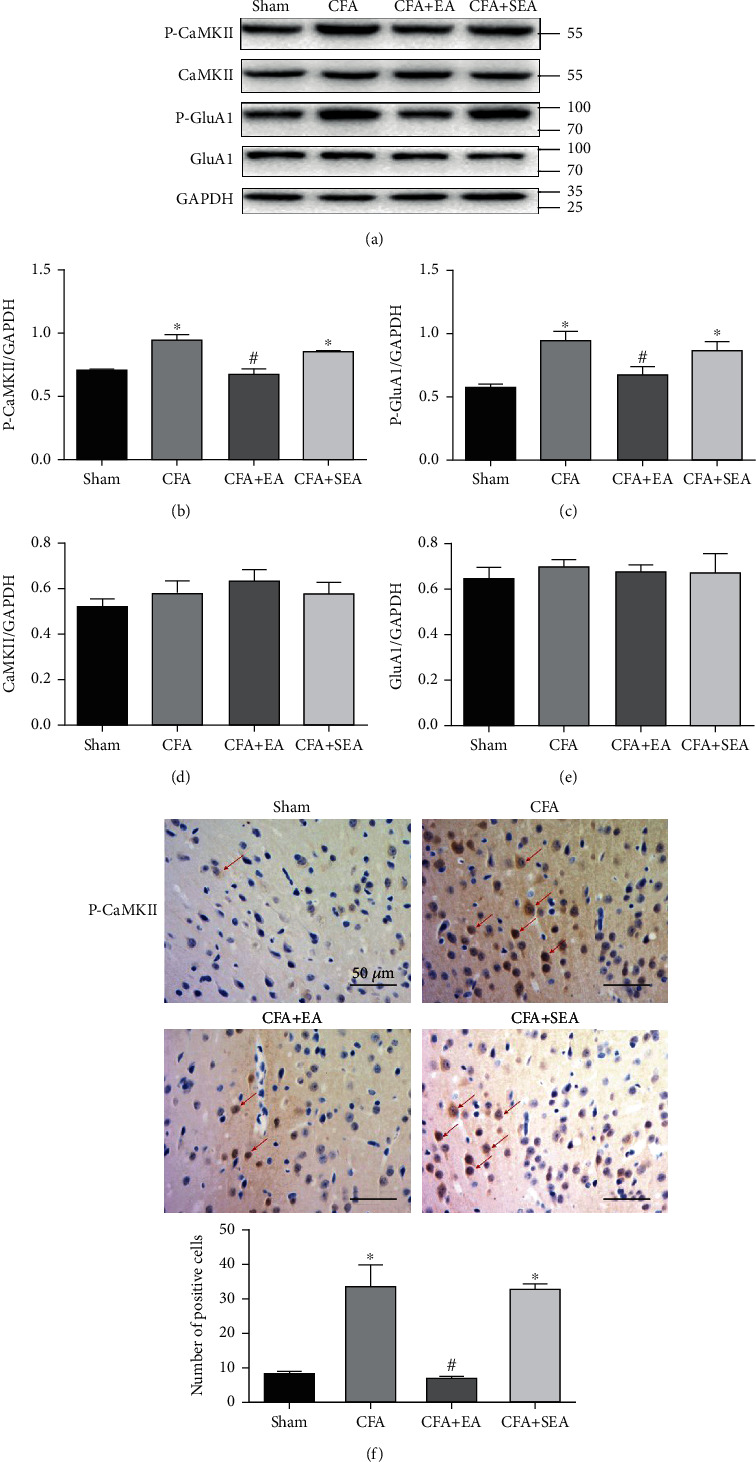
Effects of EA on the protein levels of P-CaMKII, CaMKII, P-GluA1, and GluA1. (a) Representative western blot images of P-CaMKII, CaMKII, P-GluA1, and GluA1. (b–d) Statistical analysis of P-CaMKII, CaMKII, P-GluA1, and GluA1. (f) Immunohistochemical results and statistical analysis of P-CaMKII. Scale bar = 50 *μ*m. Results represent the mean ± SEM (*n* = 4/group). ^∗^*P* < 0.05 vs. sham group; ^#^*P* < 0.05 vs. CFA group.

The ACC plays a key role in predicting pain stimuli, pain perception, and pain regulation [36]. To determine whether P-CaMKII and P-GluA1 expressions in the ACC were associated with EA analgesia, we extracted ACC tissue from the mice on the third day when the anti-inflammatory pain EA effect was most obvious. Subsequently, we determined protein expression in the three groups through western blot analysis. First, we observed CaMKII and P-CaMKII expressions in the ACC (Figure 2(a)). Compared with the Sham group, the CFA group has significantly increased P-CaMKII levels in the ACC at day 3 post-CFA injection (Figure 2(b)). Compared with the CFA group, the CFA + EA group had significantly reduced P-CaMKII levels (Figure 2(b)). This indicated that sham acupuncture did not affect P-CaMKII levels. GluA1 is among the AMPA receptor subunits; moreover, GluA1-Ser831 is the CaMKII target phosphorylation site [37]. We observed GluA1 and P-GluA1 expressions in the ACC (Figure 2(a)). Compared with the Sham group, the CFA group had significantly increased P-GluA1 levels in the ACC at day 3 post-CFA injection (Figure 2(c)). Compared with the CFA group, the CFA + EA group had significantly reduced P-GluA1 levels (Figure 2(c)). There was no significant difference in the CaMKII and GluA1 levels between groups (Figures 2(d) and 2(e)). Immunohistochemistry staining results confirmed the western blot analysis results (Figure 2(f)). These experiments demonstrate that EA can inhibit P-CaMKII and P-GluA1 levels.

### 3.3. EA Increases pCaMKII-PICK1 Complex Formation

**Figure 3:**
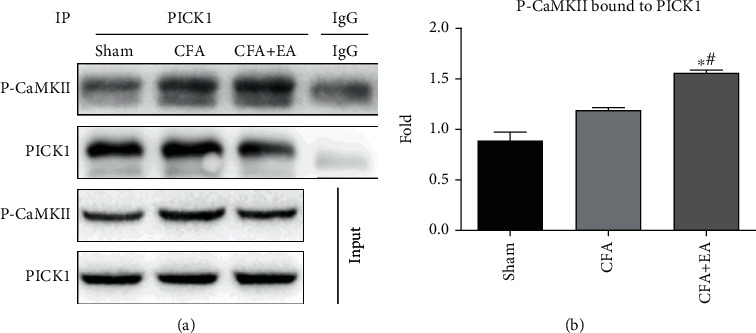
Effects of EA on the formation of pCaMKII-PICK1 complex. (a) Representative western blot images of P-CaMKII and PICK1 by CO-IP. (b) Statistical analysis of pCaMKII-PICK1 complex. Results represent the mean ± SEM (*n* = 4/group). ^∗^*P* < 0.05 vs. sham group; ^#^*P* < 0.05 vs. CFA group.

Previous studies have shown that the catalytic domain of CaMKII is colocalized with the PICK1 BAR domain in heterologous cells and neurons [31]. We previously reported that PICK1 is involved in the EA analgesic effect [32]. However, whether PICK1 and P-CaMKII can form a complex and the EA effect on this complex remains unclear. Using coimmunoprecipitation, we confirmed that P-CaMKII could interact with PICK1 to form a C-P complex (Figure 3). Compared with the sham group, coimmunoprecipitation quantitative analyses showed that the CFA + EA groups had significantly increased pCaMKII-PICK1 complex. Compared with the CFA group, the CFA + EA group had significantly increased C-P complex levels. These findings indicate inflammatory pain increased C-P complex levels, which were further increased by EA. This indicates that P-CaMKII is involved in EA analgesia as C-P complex form.

### 3.4. P-CaMKII Is Involved in the EA Analgesic Effect

**Figure 4:**
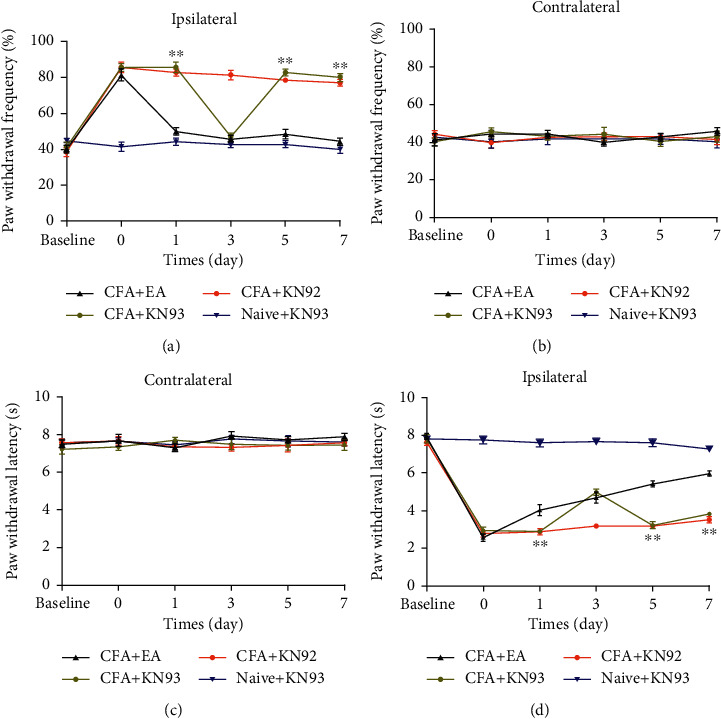
Effects of inhibitor on mechanical and thermal pain threshold (*n* = 7/group). KN93 was given on the third day of CFA injection. The paw withdrawal threshold was tested at base, 1 day, 3 days, 5 days, and 7 days. (a) The paw withdrawal frequency of the CFA + KN93 group was increased after KN93 injection (3 days), compared with the CFA + EA group on the ipsilateral side. KN92 has no effect on paw withdrawal frequency. (c) The paw withdrawal latency of the CFA + KN93 group was increased after KN93 injection (3 days), compared with the CFA + EA group on the ipsilateral side. KN92 has no effect on the paw withdrawal latency. (b, d) No difference among the 4 groups on the contralateral side. ^∗^*P* < 0.05, ^∗∗^*P* < 0.01 vs. CFA + EA group.

**Figure 5:**
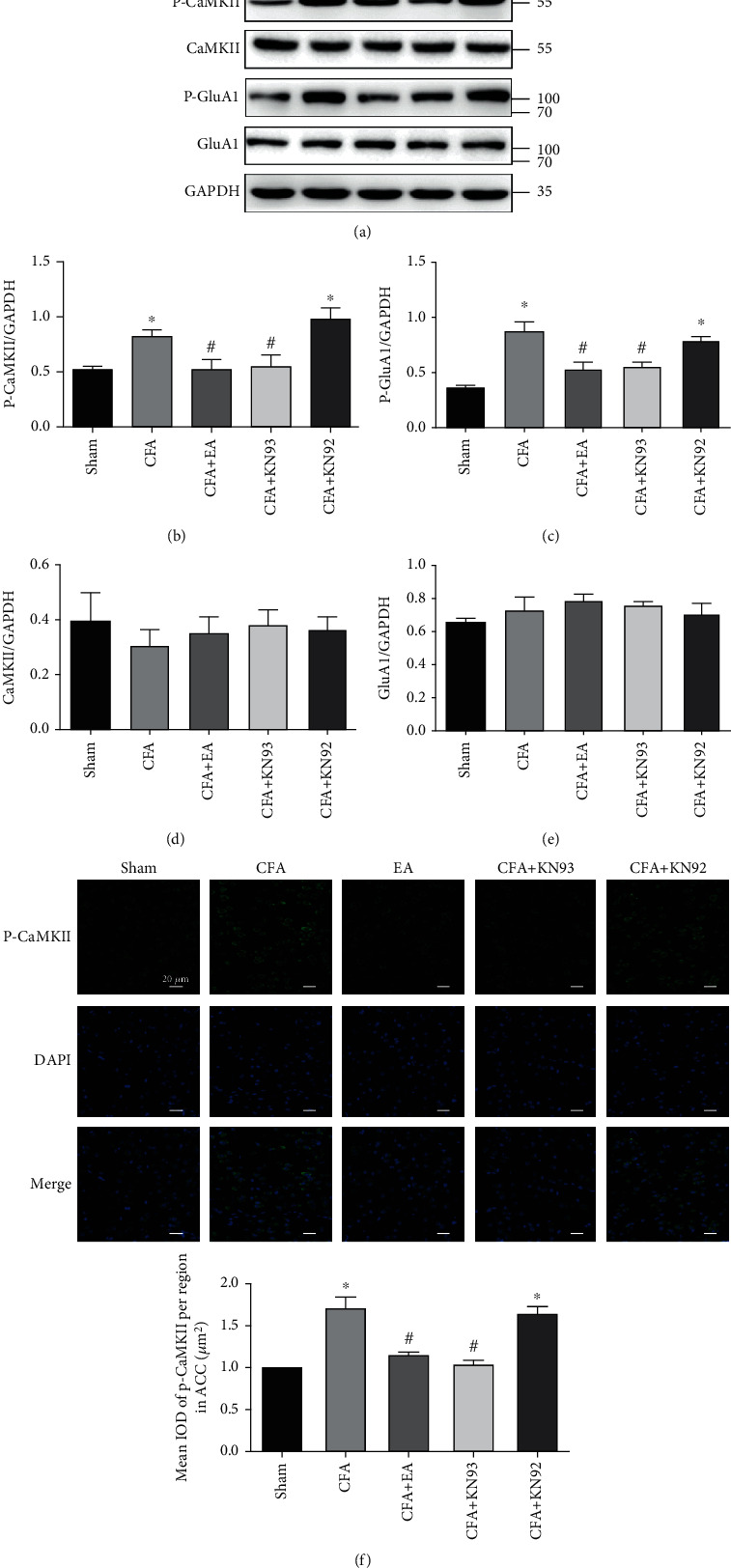
Effects of inhibitor on the protein levels of P-CaMKII and P-GluA1. (a) Representative western blot images of P-CaMKII, CaMKII, P-GluA1, and GluA1. (b–d) Statistical analysis of P-CaMKII, CaMKII, P-GluA1, and GluA1. (f) Immunofluorescence results and statistical analysis of P-CaMKII. Scale bar = 20 *μ*m. Results represent the mean ± SEM (*n* = 4/group). ^∗^*P* < 0.05 vs. sham group; ^#^*P* < 0.05 vs. CFA group.

We assessed whether EA-induced inflammatory pain reduction involved modulation of P-CaMKII expression in the ACC. Here, we used KN93, a selective CaMKII inhibitor, and KN92, an inactive KN93derivative. KN93 pretreatment and posttreatment has been shown to inhibit mechanical allodynia and hyperalgesia [19, 38]. First, we assessed the behavioral changes after KN93 use. We administered an intraventricular KN93 injection 3 days after CFA injection and measured the mechanical and thermal pain threshold after 2 hours. Similar to previous reports, posttreatment with KN93 increased the mechanical and thermal pain threshold and induced similar behavior like that in the CFA + EA group (Figure 4). Compared with the CFA group, the CFA + KN93 group significantly reduced P-CaMKII and P-GluA1 levels (Figures 5(a)–5(c)). The CFA + KN92 group had no significant changes in the P-CaMKII and P-GluA1 levels. These findings indicate that P-CaMKII in the ACC is involved in the inflammatory pain process and that EA inhibits P-CaMKII, which, in turn, downregulates P-GluA1 and alleviates inflammatory pain. Furthermore, KN93 could simulate the EA effect. Immunofluorescent staining results confirmed the western blot analysis results (Figure 5(f)).

## 4. Discussion

Chronic inflammatory pain is a serious, clinically common problem that is caused by noxious stimulation. Moreover, it has severe economic consequences for families and society. It usually manifests as persistent nociceptive hypersensitivity, including hyperalgesia at the injury site and adjacent tissues, which results from a significantly reduced pain threshold [39]. Acupuncture is a worldwide used ancient therapeutic method with a clinically confirmed analgesic effect [37, 40]. EA is an improvement of the traditional manual acupuncture method that is widely clinically used [41]. Therefore, there is a need to determine the EA analgesic mechanism to better apply it to pain treatment.

Using a CFA-induced chronic inflammatory pain model, we observed increased P-CaMKII and P-GluA1 levels in the ACC. Moreover, we observed that intraventricular injection of the P-CaMKII inhibitor KN93 reversed the nociception behavior induced by CFA. KN93 is considered a selective CaMKII inhibitor. Previous research indicates that KN93 binds to Ca2^+^/CaM and not to CaMKII, thereby disrupting the interaction between Ca2^+^/CaM and CaMKII and inhibiting the activation of CaMKII [42]. KN93 also blocks K^+^ channels. Hegyi et al. found an effect of KN93 on the delayed rectifier potassium current (IKr) in the ventricular myocytes from rabbit and guinea pig hearts. Their research indicates that KN93 has a direct inhibitory effect on IKr that is not mediated via CaMKII [43]. EA treatment decreased P-CaMKII and P-GluA1 levels in the ACC to exert an analgesic effect, which is consistent with previous studies on the spinal level [44].

The ACC plays a key role in predicting pain stimuli, pain perception, and pain regulation [45]. Previous studies have investigated the cellular and molecular mechanisms of chronic pain induction and persistence in rodent animal models. They have reported synaptic plasticity in many anatomical structures involved in pain regulation, including the spinal dorsal horn and cerebral cortex with synaptic plasticity in the ACC being the most studied [46, 47]. Studies have shown that after ACC lesions, the antinociceptive effects of contralateral EA were completely abolished. Therefore, an intact ACC is essential for contralateral but not ipsilateral EA [16]. CaMKII plays an important role in synaptic plasticity and LTP [18]. There are some similarities between synaptic plasticity and LTP, which suggests that pain and memory may have similar mechanisms [48]. Therefore, it is necessary to study the effect of CaMKII on pain treatment. Luo et al. reported that pretreatment with KN93 could prevent CFA-induced mechanical and thermal hyperalgesia while posttreatment with KN93 reversed CFA-induced pain behavior in mice. This indicates that CaMKII inhibition in the spinal cord can reduce CFA-induced inflammatory pain [19]. Moreover, subcutaneous formalin injection into the hind paws induces inflammatory pain via CaMKII is activation in the hippocampus, which is alleviated by intraventricular injection of KN93 [49]. This suggests that CaMKII is involved in pain development. The effect of CaMKII in the ACC on inflammatory pain remains unclear. We observed increased P-CaMKII expression in the ACC after CFA-induced inflammatory pain. Similar to the EA effects, intraventricular KN93 injection reduced P-CaMKII levels and normalized nocifensive behavior. Studies have reported increased excitatory postsynaptic currents in ACC neurons in the CFA-induced inflammation pain model. Consistent with these previous findings, we observed an increased amount of AMPA containing GluA1 while those containing GluA2 or GluA3 did not significantly change [24]. The deletion of GluA1, rather than GluA2, has been shown to significantly reduce periphery injury-induced Fos activation in the ACC and spinal dorsal horn, as well as the behavioral response [26]. Chen et al. further confirmed a periphery injury-induced increase in the postsynaptic GluA1-containing receptors [50]. In our study, the CFA group had significantly increased P-GluA1 expression compared with the Sham group, which was reduced by EA treatment intraventricular KN93 injection.

We previously showed that the ICA69-PICK1 complex was involved in the EA analgesic effect, which disappeared after PICK1 knock out [32]. This indicates that PICK1 plays a significant role in EA treatment. PICK1 is the substrate of CaMKII*α*, which can phosphorylate and regulate the function of PICK1 [51]. A previous study has shown that the catalytic domain of CaMKII is colocalized with the PICK1 BAR domain in heterologous cells and neurons [31]. In the present study, coimmunoprecipitation showed that P-CaMKII could interact with PICK1to form a C-P complex, which was increased by EA treatment. This indicates that P-CaMKII is involved in EA analgesia as the C-P complex. We found that EA inhibited P-CaMKII expression and increased C-P complex formation. This phenomenon could be attributed to the GluA2 subunit. CaMKII has been shown to stimulate GluA2 transport to the plasma membrane through the PICK1-CaMKII complex. Inhibiting CaMKII activity or internally stored Ca^2+^ release can inhibit Golgi's endogenous GluA2 maturation [31]. It has been shown that EA promotes GluA2 transport to the membrane, which reduces inflammatory pain [32]. More C-P complexes may be required to assist GluA2 maturation and transport.

**Figure 6:**
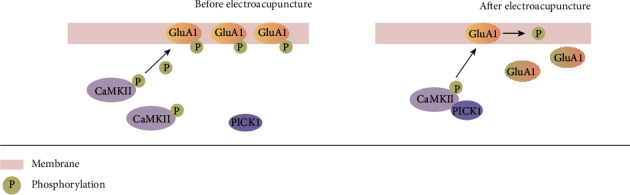
The antihyperalgesic mechanism of EA. EA treatment increases the amount of pCaMKII-PICK1 complexes. This prevents GluA1 phosphorylation, ultimately producing an anti-inflammatory pain effect.

In conclusion, our results suggest that EA exerts an antihyperalgesic effect on CFA-induced inflammatory pain by regulating GluA1 phosphorylation through pCaMKII-PICK1 (Figure 6). Our findings could be useful in identifying a novel target for EA analgesia.

## Data Availability

The data used to support the findings of this study are included within the article.

## Additional Points

*Significance*. This study is aimed at investigating the role of P-CaMKII and P-GluA1 of the ACC in a mouse inflammatory pain model. We believe that our study makes a significant contribution to the literature because it presents strong findings that demonstrate that the EA analgesic effect involves the inhibition of CaMKII-GluA1 phosphorylation in the ACC and that P-CaMKII is involved in EA analgesia as the pCaMKII-PICK1 complex.
